# Digital Design of Medical Replicas via Desktop Systems: Shape Evaluation of Colon Parts

**DOI:** 10.1155/2018/3272596

**Published:** 2018-11-14

**Authors:** Michele Bici, Valerio Cardini, Marco Eugeni, Robinson Guachi, Fabiano Bini, Francesca Campana, Franco Marinozzi, Paolo Gaudenzi

**Affiliations:** Dipartimento di Ingegneria Meccanica e Aerospaziale, Sapienza Università di Roma, via Eudossiana 18, Roma 00184, Italy

## Abstract

In this paper, we aim at providing results concerning the application of desktop systems for rapid prototyping of medical replicas that involve complex shapes, as, for example, folds of a colon. Medical replicas may assist preoperative planning or tutoring in surgery to better understand the interaction among pathology and organs. Major goals of the paper concern with guiding the digital design workflow of the replicas and understanding their final performance, according to the requirements asked by the medics (shape accuracy, capability of seeing both inner and outer details, and support and possible interfacing with other organs). In particular, after the analysis of these requirements, we apply digital design for colon replicas, adopting two desktop systems. The experimental results confirm that the proposed preprocessing strategy is able to conduct to the manufacturing of colon replicas divided in self-supporting segments, minimizing the supports during printing. This allows also to reach an acceptable level of final quality, according to the request of having a 3D presurgery overview of the problems. These replicas are compared through reverse engineering acquisitions made by a structured-light system, to assess the achieved shape and dimensional accuracy. Final results demonstrate that low-cost desktop systems, coupled with proper strategy of preprocessing, may have shape deviation in the range of ±1 mm, good for physical manipulations during medical diagnosis and explanation.

## 1. Introduction

The prospect of manufacturing complex shape is the key factor capable of bridging additive manufacturing (AM) with medical applications [1]. AM represents an ideal choice for a small-scale customized production, as the production of patient-specific objects is. In fact, in contrary to conventional manufacturing techniques, variations from the nominal design may have a reduced impact on the AM planning and related costs [2]. This point holds not only in medical applications but also in all cases that need a high level of customization, as, for example, in space applications [3]. To date, medical researchers and clinicians have had limited access to the process knowledge of 3D printing technologies. Now, this is rapidly changing, and many surgery and radiology practitioners are starting their own 3D printing labs. The knowledge of advantages and limitations of the various 3D printing technologies is a key factor to accomplish successful investment and to extend 3D printing into medical field. In the literature, many works address AM application for medical purposes, as described in [1]. They range different areas from preoperative models up to implants and surgical tools and aids.

AM is currently applied in maxillofacial and orthopedic, both for customized implants and tools and coronary surgery [4, 5]. In [6], it is used for the rapid delivery of the fractured skull model. The effectiveness of the use of this model as a preoperative guide is shown. Time-consuming operative actions for the reduction of fractures can be lessened if the preoperative model of the fractured jaw is provided. In [7], AM is used for a new approach to reduce the eye cavity fracture. Usual procedure provides the surgeon manually shaping metallic plates according to what he finds during the surgery. The proposed procedure allows the fast delivery of skull model of the patient. Based on this, an enhanced implant is designed, allowing for better integration with neighboring bones. Results show an improved position of the artificial eyeball along with a reduced surgical time, thanks to a better surgical planning. It is also proved that the quality of the 3D models of the skull plays a crucial role since shape inaccuracy may give ineffective implant. In [8], geometric modeling issues are addressed to measure and reproduce fragments of a skull. In [9], AM has been used for getting liver models. Based on these, surgeons get clearer ideas about possible surgical cuts in liver transplantation operations, helping in reducing the risk for the donors. These considerations have been effectively and successfully implemented in liver transplantation operations. High accuracy of the replicas is shown by comparing them with real livers through visual inspections and measurements taken during the operations. Again, model accuracies are of great importance. No reliable surgical cuts could be planned with inaccurate models. In [10], a workflow for digital models of fetal faces is proposed to help diagnosis of cleft lip disease and to investigate affective effects on parents.

Among AM technologies, fused filament fabrication (FFF) is currently the most widespread, in part due to the expiration, in 2007, of the initial patent from Stratasys, the company that invented the technology with the name of fused deposition modeling (FDM). This, along with the simplicity of the system, allowed the proliferation of several companies providing low-cost systems, also called desktop systems [11]. To provide a numerical example, we can just consider that, in 2015, the ratio between expensive commercial systems and low-cost desktop systems (that means below $5000) was about 1 : 20. In addition to cost considerations, desktop systems can be operated with major adaptability. Selected material can be chosen among those from different vendors. Very limited restrictions on available setting values of the process parameters are provided. On the contrary, this freedom does not guarantee any assurance of quality by the vendor, as it usually happens for more expensive solutions, which are properly restricted to guarantee component characteristics and stability in terms of material, size, and shapes.

Desktop systems seem to be advisable for medical replicas that can be adopted as tools for managing surgical planning or for better understanding specific patient-related aspects. They can be able to translate, easily and with low costs, 2D DICOM (Digital Imaging and COmmunications in Medicine) analysis into a physical *replica* (also called *mock-up* [12]), helping a proper perception of actual shapes and lengths. In [13], medical replicas of skull and mandible, made by professional FDM systems, are investigated from the metrological point of view. Applying the comparison between digital models and replicas of different genders and age, authors declare outstanding accuracy (overall absolute average deviation of 0.24%) of FDM in comparison with other rapid prototyping techniques. In [14], the performance of a low cost FDM system for the delivery of 3D models of mandibles are presented and discussed. Even though the performances are satisfying, no discussion is presented on the use of the system, in terms of digital design.

Multiple studies have documented that medical replicas can be produced with spatial errors of less than 1 mm [15–17]. Generally speaking, shape and dimensional inspection are relevant for medical applications due to the necessity of

- classification [18]
- quantification of gravity and evolution of malformations [19] and
- interactions among organs and tools.

AM technology involves different sciences and almost every aspect that has been modeled in literature [20, 21]. Nevertheless, also due to the large number of technologies, process knowledge and robustness are not mature yet. This lack of knowledge translates uncertainties in the manufactured object and standard tolerance controls that are still missing.

The digital design workflow asks for a data preprocessing that requires specific skills concerning 3D modeling from DICOM; tessellation for slicing; and definition of process parameters according to functional and quality requests [22].

Part of these topics asks for new oriented approaches and solutions, since they are related to a “new” technology [23]. Although these steps are becoming common skills in the engineering field, they are not always available in the medical field, yet. For these reasons, this paper discusses the most critical aspects (hardware, software, and process parameters) to be accounted for implementing digital design of medical replicas through desktop systems. After their presentation, a test-case consisting of part of a colon, from sigmoid up to the rectum, is presented. It aims at providing an experimental overview of the process and a quantitative evaluation of replicas accuracy, through experimental shape acquisitions made by a structured-light system. For the experimental part, two low-cost desktop systems have been applied and compared, a Sharebot and a Tevo-Little Monster.

According to this aim, in Section 2, we present the general workflow of digital design in the respect of final accuracy; then, in Section 3, we discuss the requirements set by the medical application; finally, we present and discuss the application made and the related experimental results.

## 2. Digital Design Workflow and Accuracy

Regardless of a specific AM process, in a digital design workflow, we can distinguish the following steps [24]:

- preprocessing that concerns with 3D model setup as suitable STL file
- CAM setup of the slicing and processing that consist in the manufacturing of the component
- postprocessing that pertains to the removal of the component from the manufacturing table and also the removal of the outer supports and other operations necessary to guarantee final shape and roughness (gluing of separated parts, surface finishing, etc.).

**Figure 1:**
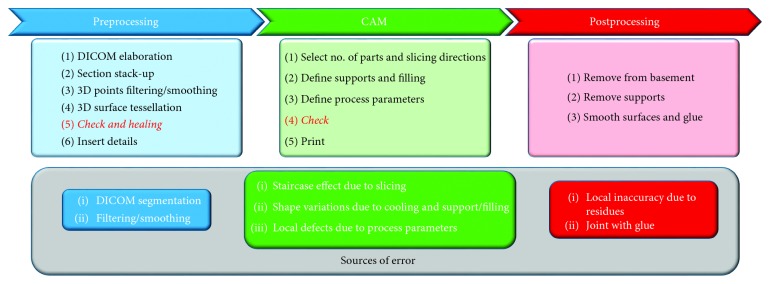
Digital design workflow for medical replicas and related sources of error.

Each of these steps has its own workflow that can be specialized according to the specific field of application. In Figure 1, some further details have been addressed according to application for medical replicas.

The preprocessing starts from the capture of the area of interest from the medical imaging (DICOM). They can be obtained from different technologies, such as computed tomography (CT), positron emission tomography (PET), X-ray, ultrasound, etc. DICOM images are bidimensional images of transversal sections of the body. These sections are generally taken with a much variable range of steps (we have assumed steps of 3 mm, that represent, in our specific case, a good compromise between time consumption and accuracy for wide areas scanning). They have to be processed by segmentation to isolate the area of interest from the rest of body's sections. Then, the set of sections that pertain to the volume of interest (e.g., part of an organ) are stacked up to obtain the 3D cloud of points, filtered, and/or smoothed so that a suitable tessellation of the 3D model can be achieved. In the majority of cases, especially with X-ray, specific algorithms have to be employed [25]. Errors, during this step, may be wrong segmentation due to misclassified pixels; lack of accuracy due to larger scan step; and 3D cloud of points with holes, thus inaccurate tessellation. It has to be checked according to regularity (no intersecting or non-manifold triangles should be present) and integrity. If not, proper healing functions has to be applied, such as optimization of shape triangles and filling of holes. Absence of this step may result in the incorrectly manufactured objects, or error break of the AM process [2]. After the check, a modeling step can be taken into account if specific functionalities must be provided, for example, the capability of open part of the organ for inner inspection.

CAM setup and processing (CAM) includes *slicing* that pertains to the selection of the layer direction and the subdivision of the 3D model into a set of sections, called slices, one over the other. In this step, also the necessity of splitting the replica into parts must to be evaluated. It can be due to the presence of opened details, which allow inspection or visibility, or due to other manufacturing constraints (e.g., volume of the printing camera). The result of the slicing is an approximated 3D object, given by the stacking of the 2D slices. The height of these is equal to the user-defined *layer-thickness* parameter. External surfaces not aligned with the vertical direction will inevitably exhibit the so-called *staircase* effect. Layer thickness influences this approximation. Normal orientation of the slicing represents the orientation of the process. It also has effects on the necessity of infill and supports, to avoid collapse of undercuts or unsupported slices not solidified yet. Supports reduce the final quality of the surfaces and ask for postprocessing work, to remove them carefully.

Concerning the definition of the process parameters, it is related to the adopted technology. FFF allows to manufacture the slices by extruding fused polymeric filament through a nozzle. Commonly adopted materials are acrylonitrile butadiene styrene (ABS) and polylactic acid (PLA), in the form of loose filament coil. They represent not-expensive solutions, mainly for rapid prototyping [2, 26]. As fundamental component of any FFF hardware, the liquefier nozzle (generally with diameters from 0.2 to 2 mm) allows to melt the filament by reaching the related temperatures (in the range of 100°–200°C, for ABS and PLA). Once liquefied, the polymer pours out through the nozzle, thanks to the pressure applied to the solid filament. It is pushed by the pinch rollers mechanism. The polymer is deposited on a printing plate or on a previously built layer. Polymer melt solidifies as heat is lost to the surrounding environment. The head through which material is poured out can move all along the printing bed plane, i.e., *x*-*y* directions, as this can be moved independently along height direction (*z* direction). Through this mechanism, a 3D object can be printed. For a deeper discussion of the process, along with state-of-the-art science modeling, the reader can refer to review papers [20, 21]. One of the major drawbacks of this setup is related to the temperature gradients during depositions. If they occur suddenly, not uniform cooling conditions may arise inside the manufactured part and thus residual stresses may occur (shrinkage). To reduce shrinkage, chamber temperature should be guaranteed. For desktop systems, which commonly do not have a closed and controlled chamber, a heated printing plate may help to achieve better conditions. In addition, a correction factor may be applied during the CAM preprocessing to compensate shrinkage, as a function of the selected material. Other problems that may affect this process are the conditions of filament extrusion at the nozzle. The layer thickness may be not uniform and stable at the nominal value. It can change due to problems with the mechanism of the material supply vector up to the nozzle, or due to friction-thermal discontinuities in the nozzle. These aspects may be of utmost importance for desktop systems, since they are not guaranteed or optimized in respect of all the process variables, as it happens for commercial systems [27–29].

Concerning the postprocessing step, it is necessary to remove the part from the manufacturing platform and possible supports that surround it. Surface finishing can be improved by proper operations, so that residual of the supports and staircase discontinuities are improved. They can be mechanical operation or low-cost chemical solutions such as acetone vapor bath. Chemical solutions provide to be very effective [30], but they are only applicable to certain materials. Acetone vapor bath, for example, does not work with polylactic acid (PLA).

## 3. Digital Design Requirements for Medical Replicas

Surgeons use tangible life-sized models of individual anatomy for preoperative planning, explanation of the procedure to the patient, and, as a reference, during the surgical procedure. 3D printing offers advantages over conventional manufacturing technologies. Personalized single models can be created, as needed in a clinical setting, with relatively low cost in a fairly short time frame. Medical requirements for the digital design can be summarized as follows:

1. Area of interest:
  - Selection of a single organ/fragment or more than one organ
  - Position of the interesting areas (inner area, outer area, and both inner and outer areas)
2. Providing pins and positioning elements for exhibition:
  - For mounting parts among them
  - For opening/closing interesting areas
3. Good dimension and shape accuracy

**Figure 2:**
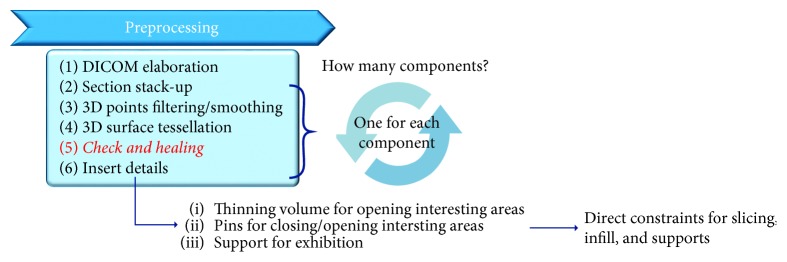
Details of the preprocessing substeps for medical replicas.

These requirements constraint the preprocessing and the CAM steps as depicted in Figure 2.

More in detail, the first requirement directly derives from DICOM segmentation, and it may have consequences on the material selections (if multiple organs are replicated, multiple materials/colors may be selected [9]). Presence of details inside the organ asks for a replica that has to be opened. It impacts the replica modeling step, asking for (a) modeling the separation of the part in coincident fragments; and (b) modeling the closing pins.

The necessity of parts that have to be opened impacts also the manufacturing step, since in case of complex shapes, it usually asks for supports during slicing. For fast prototyping, supports have to be minimized to avoid postprocessing problems. As a consequence, the shape of the surface to be opened is defined through a criterion that provides a trade-off between the maximization of the inner area dimensions, for visibility and maneuverability, and the minimization of supports, for finishing and aesthetics.

The second requirement (providing pins and positioning elements for exhibition) asks for adding surface details to be embedded in the tessellation of the interesting area, or external volume to be built to maintain the replica fixed. In any case, it may introduce local modification of the model with consequences on the process setup. Positioning elements may be also designed separately from the replicas.

**Figure 3:**
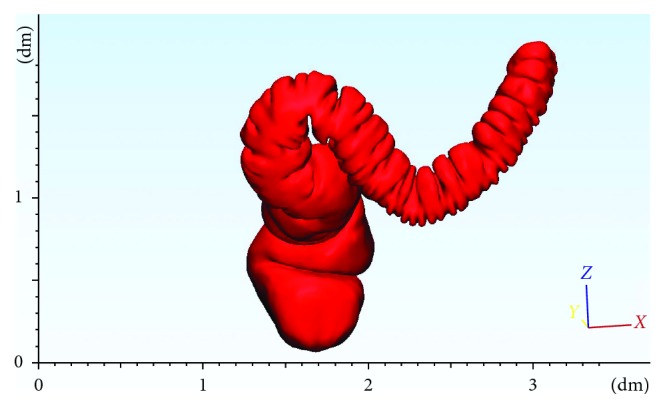
3D model of a descendent/sigmoid colon and rectum after DICOM segmentation and 3D point surface reconstruction.

Complex shapes of medical replicas are typical free-form surfaces, characterized by curvatures in different directions and curvilinear axes. In the colon case, no transversal section is equal to any of the others in terms of lengths and morphology (Figure 3).

**Figure 4:**
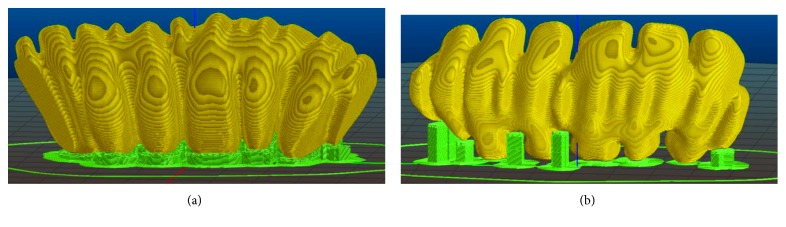
(a) Slicing simulation according to optimal direction to minimize height of the supports. (b) Slicing simulation in a different orientation.

To guarantee accuracy, the most stringent manufacturing constraint becomes the reduction of supports. Supports must be removed mechanically, and this would inevitably end up in corrupting the quality of the surface where the two structures mate [2]. In addition, mechanical action may induce cracks [31], hidden below a roughly defined surface. To avoid these problems, it is necessary that a subdivision of the axis in linear segments is made, so that each subpart may have proper slicing orientation, suitable for minimizing both staircase effect and request of supports.  Figure 4 shows this concept applied to a segment of the colon shown in Figure 3.

In Figure 4(a), its slicing (green) is simulated along the direction able to minimize the supports' height and the difficulties in their removal. In Figure 4(b), although supports are more concentrated, their heights are more relevant and protruded inside the folds, so that major difficulties may arise during removal. Obviously, in case of solution in Figure 4(a), the small area of the replica connected to the supports will be affected of bad finishing after their removal. However, in Figure 4(a) solution, postprocessing operations, in order to improve finishing, can be done more easily than in Figure 4(b) case.

**Figure 5:**
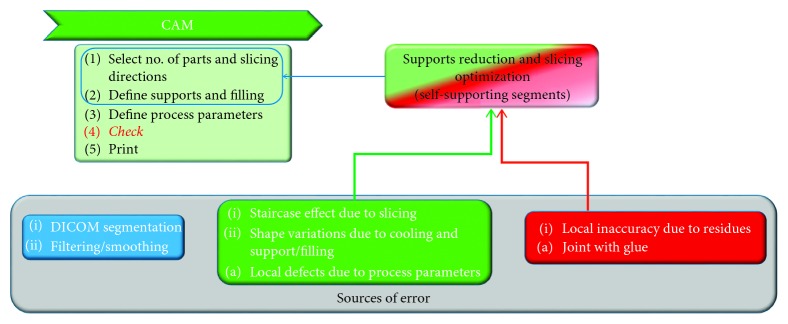
Constraints to CAM step in the digital design workflow for medical replicas.

To obtain a better surface accuracy, a longitudinal cut that breaks each segment in two parts can be defined. In this case, a proper slicing orientation means looking for the section plane, along the central axis of the considered segment, which includes the projection of the overall semivolume of the segment. By doing so, the segment will be subdivided in two subparts with minimum height and maximum in-plane surface so that the volume will be self-supporting. In this case, the division will ask for gluing the parts after the process, but it avoids rough surfaces and the necessity of postprocessing, factors that may reduce shape and dimension accuracy. The test case described in the following sections will apply this solution, to minimize postprocessing efforts. Finally, Figure 5 summarizes the concepts behind this reasoning in terms of source of error and efforts necessary in the CAM step of the digital design workflow.

## 4. Application

### 4.1. Test-Case Description

The proposed workflow has been applied to evaluate replicas of part of a colon and its final rectum (Figure 3). In the latter, abnormal growths and polyps are internally and externally visible. This justifies two different manufacturing strategies. As there is no interest in representing the internal part of the colon, this will be built as a dense part. Conversely, the final rectum will be built as a hollow part, with attention given to the polyps and abnormal growths. Their shape and position, together with the specific shape of the colon folds, are fundamental for surgeons, and, ultimately, they justify the manufacture of the medical replica. Parts have been replicated through two different desktop systems: a Sharebot and a Tevo-Little Monster, from now on, respectively, called DS#1 and DS#2.  Table 1 shows an overview of their declared plate values.

**Figure 6:**
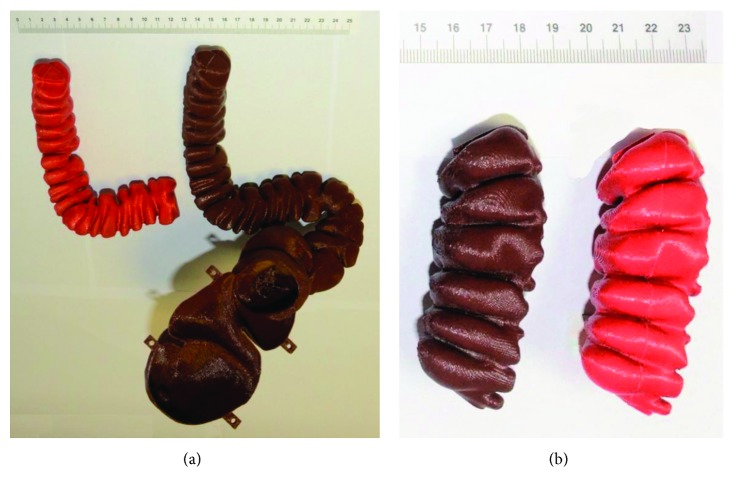
DS#1 final replicas (red) and DS#2 final replicas (brown): (a) final assembled models; (b) replications of the first segment of the sigmoid colon.

More in detail, the sigmoid colon has been replicated by DS#1 and DS#2, while the final rectum has been printed by DS#2.  Figure 6 shows the final replicas, red parts are related to DS#1 and brown to DS#2.

Adopted material, in both cases, is PLA. For DS#1, selected parameters are layer height of 0.15 mm, with the exception of the first layer (0.30 mm), starting from a filament diameter of 1.75 mm. Temperature at the nozzle was 230°C, at the plate 60°C. We choose to follow the shape for 3 perimeters in each layer, and then, fill the inner part through a honeycomb 2D structure, extruded in *Z* direction. For DS#2, filament diameter was 1.75 mm with layer height of 0.16 mm. Temperature at the nozzle was 230°C, at the plate 80°C. External perimeters were made at 40 mm/s of speed, otherwise it was higher, up to 650 mm/s for nonprintable areas. In this case, we choose to follow the shape for 4 perimeters in each layer, and then, fill, as previously, the inner part through a honeycomb 2D structure, extruded in *Z* direction. The honeycomb fill pattern has been chosen with a density of 15%. Supports are the same in both cases.

To assess the quality of the replicas, measurements have been made and compared with the 3D model. They have been carried out both through caliber and reverse engineering acquisitions, made by a structured-light commercial system (Scan in a Box-FX) that declares accuracy of 0.04 mm with a minimum resolution of 0.062 mm.

### 4.2. Preprocessing and Manufacturing

As said in Section 3, good accuracy can be obtained by reducing the outer supports and minimizing the number of single fragments. Subdivision in fragments is mainly due to the complexity of the shape, that have different planes of maximum envelop surface projection, along its axis. In addition, the subdivision procedure is necessary due to the usage of very simple desktop systems, with one single nozzle. Thus, the component to be printed and the supports, if present, must be of the same material. Due to this, the mechanical removal of supports may cause, also, fractures and breaks in the component. Otherwise, if more than one nozzle is available in the system, other removal techniques and procedures can be developed.

**Figure 7:**
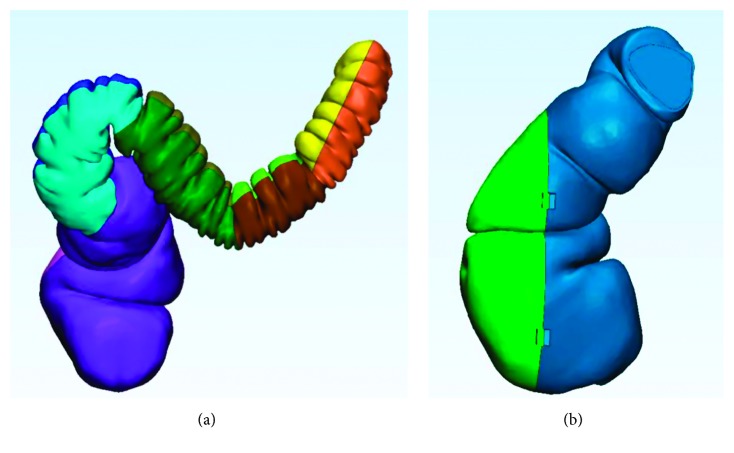
3D model subdivision (one color per manufactured part): (a) spine subdivision in segments; (b) rectum subdivision to see the inner surfaces (part to be opened (represented in green)).

Figure 7 shows, with different colors, the final fragment subdivision of the test-case. The obtained 5 segments, four for the sigmoid colon and the last one for the rectal ampulla, are the result of the localization of the medial axis and the segmentation into parts that can be built minimizing outer supports.

The four colon's segments have been separated again in two parts. This limits inaccuracies on the external surfaces, providing a planar contact area with the printing plate, not involving the external surface. In addition, the cutting planes have been selected as the plane that maximizes the normal projection of the envelop area of the part. In this way, imposing also a slicing orientation orthogonal to the cutting plane, we are able to minimize or completely avoid undercuts, especially in folds, leading to the minimization of supports.

**Figure 8:**
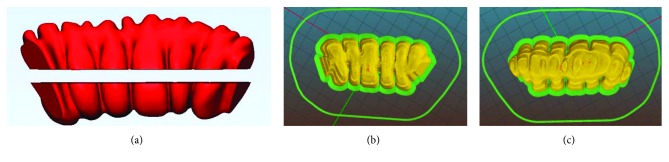
First colon segment: (a) model subdivision into two parts and (b, c) related slicing simulations.

**Figure 9:**
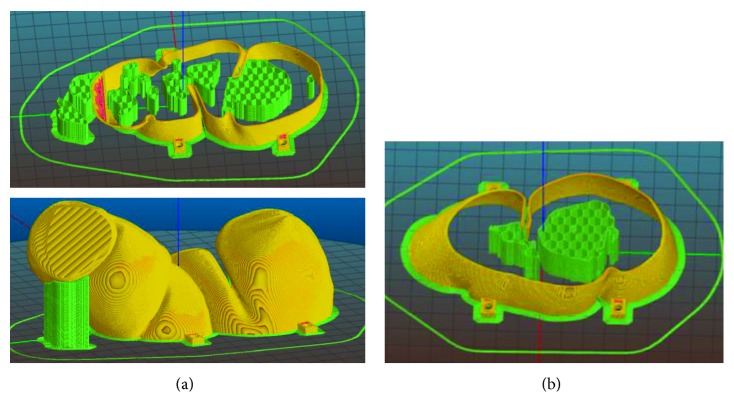
Rectum segment: (a) slicing of the part connected to the colon; (b) slicing of the part to be opened.

Figure 8 shows details of the subdivision in two parts of the first fragment of the colon at the top of the 3D model. It is clearly shown that, in this case, no supports are necessary, considering the inner volume filled by infill structure.  Figure 7(b) shows the subdivisions adopted for the final segment related to the rectum. In contrary to the colon fragments that are bulked, they are related to a thin-wall volume that has to be opened for inner inspection. As a consequence of their free-form shape, supports are necessary to avoid collapse of the inner surfaces with maximum heights from the base and of the prominent details of section, as shown in Figure 9. Also in this case, cutting plane has been found to minimize heights and thus supports, together with the necessity of opening a specific volume, according to the inner area of interest that has to be shown.

Axis analysis, fragment subdivision, and slicing orientation have been made in a CAD environment interactively. Optimization concerning the minimization of the supports has been iteratively checking the added volume of the supports.

## 5. Experimental Results and Discussion

All the planned parts resulted in a successful outcome. A quantitative comparison of the AM replicas with the tessellation obtained from the DICOM has been obtained by some reverse engineering acquisitions. They are related to the first segment in the upper part of the colon. Due to the possible light reflection on the acquired surfaces, during the acquisition with the structured-light system, the segments were covered with powder. Each segment was acquired in multiple views, aligned via embedded software (IDEA) through best fitting. After this, the cloud of points were superimposed to the 3D model and measured via distance analysis. Acquisitions consists of more than 480 × 10^3^ points that have been analyzed after a selective filtering, based on curvature analysis, which left about 91 × 10^3^ points.

**Figure 10:**
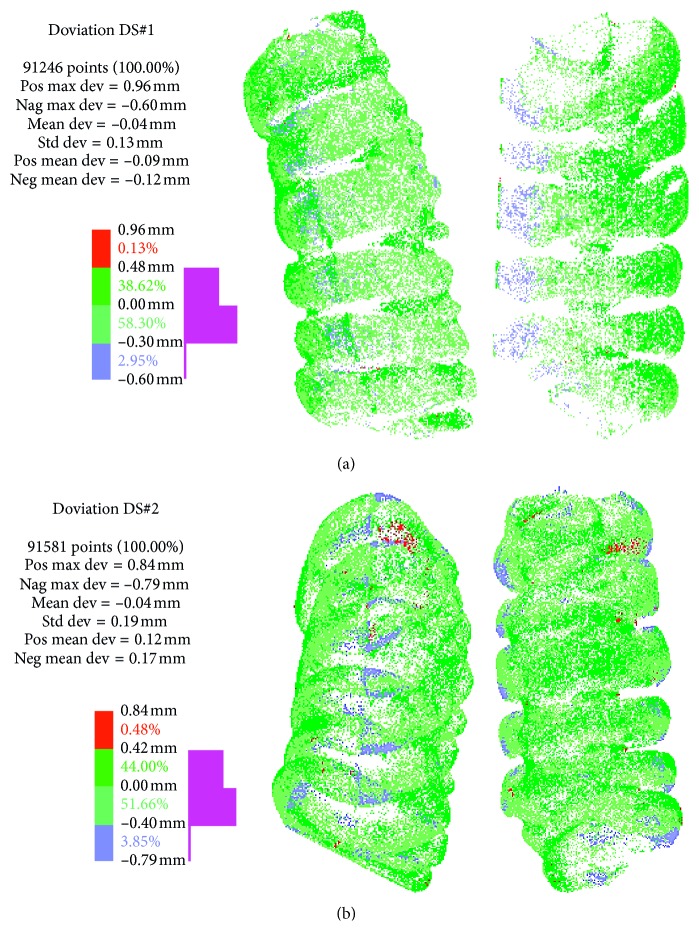
Experimental results related deviation analysis between 3D model and acquired replicas: (a) DS#1; (b) DS#2.

Figure 10 shows the results in terms of color maps. In both cases, the deviation range is less than ±1 mm. Specifically, more than the 95% of points, in each case, are included between +0.48 mm and −0.40 mm. Both in DS#1 and DS#2, the mean deviation is quite close to zero, showing a little negative value (−0.04 mm). Due to this, the shrinkage can be assumed as controlled, even though it is present in blue restricted areas (small entity). This result is consistent with that already found in the literature (value of deviation less than 0.1 mm is defined as absence or controlled shrinkage) [13].

Staircase effect and filament thickness cannot be seen due to the limit of the adopted system; otherwise, along the contour of the glued parts, the discontinuity is clearly shown in DS#2 (Figure 10(b)). While in case of DS#1, the systematic position of the blue areas together with the small values of mean deviation was achieved, let us say that this trend could be dependent on possible inaccuracies during alignment or acquisition than on the presence of shrinkage or error during the manufacturing process.

Concerning the rectum ampulla segment, the one that can be opened, a thickness evaluation, made by the caliber, has been performed. It confirms an averaged value of 1 mm, equal to that imposed in the digital design.

These experimental evidences confirm that good shape accuracy can be reached adopting low-cost desktop systems, also for very complex free-form shapes.

Taking care of minimizing support presence and avoiding severe cooling conditions of the material (e.g., increasing speed of the deposition of the external surfaces) have allowed to obtain good replicas of all the segments, without the necessity of surface postprocessing. On the contrary, the necessity of gluing the single parts cannot be reduced. Nevertheless, as shown through the experimental results, it induces errors less than 1 mm.

## 6. Conclusions

In this paper, the digital design workflow of medical replicas has been analyzed and applied to reproduce a large part of the colon. Two low-cost desktop systems, a Sharebot and a Tevo-Little Monster, have been adopted to achieve replicas, made of PLA.

The digital preprocessing of the DICOM data has been approached according to the requirements of (a) making the replica partially opened, and (b) limiting surface postprocessing due to support removal. The opened area is the rectum one, which has been obtained as a thin-walled segment, with one removable shell. The other segments, instead, are filled in by honeycomb fill-in at 15%.

Due to the complex free-form surfaces, avoiding or limiting surface postprocessing mainly means minimizing the presence of supports and thus, optimizing the slicing directions. According to this, the central axis of the overall surface has been divided into 5 segments, suitable for reproducing the related surfaces with the optimal slicing direction. The optimality is found looking for a plane that passes through the central axis of the segment and that is able to define two self-supporting volumes, so that local supports of the complex surfaces on the printing table are avoided. In order to guarantee visibility and accessibility to its inner surface, the fifth segment (related to the rectum) has to be provided as a thinned volume, so that some supports should be necessarily present.

This preprocessing step represents the most consuming part of the workflow, and although the cutting planes may be provided automatically, the overall evaluation of how many segments must be used for the division is, currently, still a manual step, based on integrated product-process skills.

After achieving all the replicas of the segments, they were glued together. The final shapes of the first colon segments, opposite to the rectum, have been digital acquired by a structured-light system. The deviation analysis from the experimental results shows shape errors lower than 1 mm, with more than the 95% of experimental points with errors less than 0.5 mm. Point's statistical distribution shows absence (or high limitation) of shrinkage effects, with an almost symmetrical distribution of the deviation (mean value smaller than 0.1 mm).

To conclude, this paper shows that desktop systems can be used as fast solutions for medical replicas, demonstrating a good level of accuracy in complex shape reproduction. In terms of easiness of workflow, geometrical design criteria have been defined to guarantee surface finishing through the reduction of supports, minimizing the postprocessing efforts. Nevertheless, without a proper automation of the processing of the 3D model to set the segments, preprocessing still represents the bottleneck for medical autonomous applications without added skills from the engineering field. As future work, algorithms to provide the automation of the process will be analyzed and developed. In addition, the research will be moved to the possibility of generalization and reapplication on other different kinds of organs.

## Figures and Tables

**Table 1:** Adopted desktop systems: declared plate values.

	Desktop system DS#1	Desktop system DS#2
Model	Sharebot	Tevo-Little Monster
Printing size	250 mm × 220 mm × 200 mm	340 mm × 340 mm × 500 mm
Material	PLA-S, nylon-carbon, thermoplastic polyurethane	ABS, flexible PLA, HIPS, nylon, PVA
Minimum layer thickness	0.05 mm	0.05 mm up to 0.4 mm
Heated printing plate maximum temperature	90°C	80°C

## Data Availability

The reverse engineering data and manufacturing parameters used to support the findings of this study are included within the article. Input data and all other data used to support the findings of this study are available from the corresponding author upon request.
